# Hemophagocytic Lymphohistiocytosis (HLH) Flare Following the COVID-19 Vaccine: A Case Report

**DOI:** 10.7759/cureus.56773

**Published:** 2024-03-23

**Authors:** Rabia Iqbal, Aemen S Bazaz, Amina Jafar, Taimoor Bajwa, Kanchan Devi, Joshua A Wilson, Ana Colon Ramos, Samridhi Sinha

**Affiliations:** 1 Internal Medicine, The Brooklyn Hospital Center, New York, USA; 2 Internal Medicine, Shifa International Hospital Islamabad, Islamabad, PAK; 3 Internal Medicine, Shaukat Khanum Memorial Cancer Hospital and Research Centre, Lahore, PAK; 4 Hematology and Oncology, The Brooklyn Hospital Center, New York, USA

**Keywords:** covid-19, elevated ferritin level, vaccine, inflammatory response, hemophagocytic lymphohistiocytosis (hlh)

## Abstract

Hemophagocytic lymphohistiocytosis (HLH) is a rare hyperinflammatory state that leads to overactivation of the immune system due to underlying disease. It can lead to multiorgan failure and death if not treated properly. HLH after vaccination is rare but has been reported in a few cases. Here, we present a case of a patient who developed HLH after she received the COVID-19 vaccine. Workup was done to find the underlying cause of her symptoms. However, as all investigations yielded negative results, the close temporal association with the COVID-19 vaccine suggested that the vaccine might have been the causative factor. She was treated appropriately with steroids and discharged with follow-up appointments. The exact pathogenesis of HLH caused by COVID vaccine is unknown, but it is likely related to the inflammatory response caused by the vaccine in the body. The treatment of HLH depends on the treatment of underlying conditions causing the HLH. Very few cases have been reported in the literature regarding HLH caused by COVID vaccine. Immediate recognition and treatment are imperative for improved prognosis and improved chances of survival.

## Introduction

Hemophagocytic lymphohistiocytosis (HLH) is a disorder characterized by a hyperinflammatory state and tissue damage caused by overactivation of the immune system due to an underlying disease or genetic defect. It is a syndrome that can be life-threatening and requires immediate medical intervention. HLH is a disease of the mononuclear phagocytic system, which involves an inflammatory response to an underlying disease or illness. There are multiple presentations of the disease, and it often leads to end organ failure. The diagnosis of HLH is challenging, too, as it is often not diagnosed on initial bone marrow biopsy but requires serial biopsies [1]. The lack of awareness and guidelines in adults leads to delays in diagnosis and treatment, which eventually leads to organ failure and death. The COVID-19 vaccine has the potential to cause inflammatory responses in the body. Although it has reduced COVID-19 infection-related mortalities, a few serious hematological complications have been reported, such as multisystem inflammatory response [2] and thrombotic immune thrombocytopenia [3].

A few case reports have been published that present cases where patients developed HLH after the COVID-19 vaccine. Here, we present a case of a 28-year-old female who was previously diagnosed with HLH and came to the hospital with HLH exacerbation after she received the COVID-19 vaccine.

## Case presentation

In this study, we report a 28-year-old female who had been diagnosed who came to the ED with complaints of sore throat, generalized body aches, severe joint pain, weakness, rash, and inability to walk for three days.

On the physical exam, temperature was 97.8, heart rate was 104, blood pressure was 118/76 mm of hg, and respiratory rate was 20 breaths per minute. White plaques were seen on the tongue and posterior oropharynx with mild erythema, +1 pitting edema in BL LE extremities, and rash present all over the skin. The rash was punctate, non-maculopapular, and slightly raised on the upper and lower extremities.

A review of the system was remarkable for chest pain, shortness of breath, joint pain, weakness, inability to walk, non-itchy rash, and joint pain. Family history was significant for celiac disease in her mother. She revealed that she had received COVID vaccine almost two weeks before the onset of symptoms. Initial differential diagnoses included systemic lupus erythematosus, rheumatoid arthritis, cryoglobulinemic vasculitis, Guillain-Barre syndrome, and HLH.

**Figure 1:**
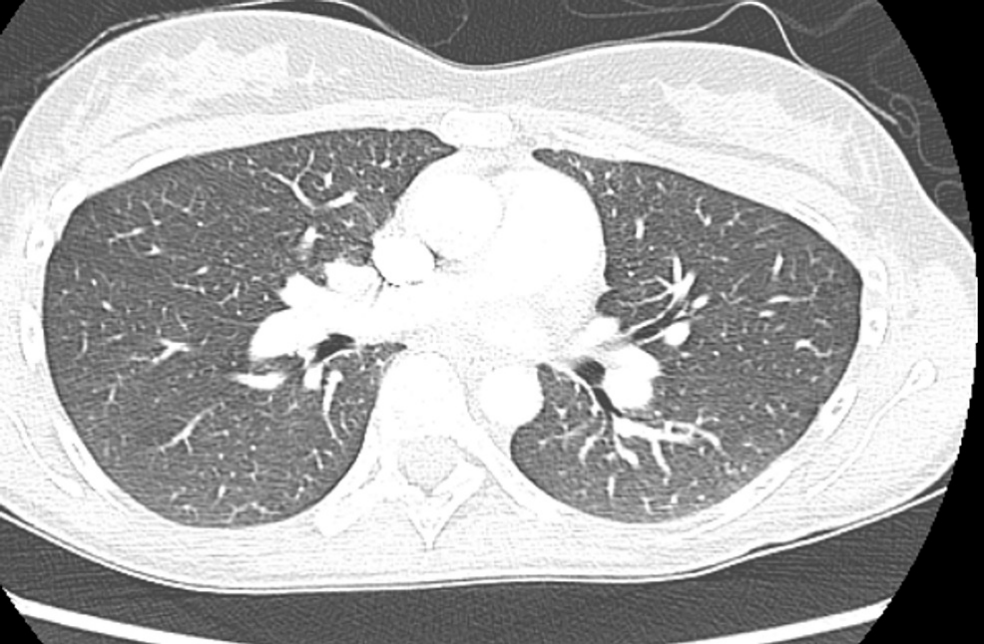
CT Angiography of the chest did not show any central, lobar, segmental, or proximal sub-segmental pulmonary embolism

Images included a chest X-ray, which did not show any acute pathology. CT angiogram of the chest was negative for pulmonary embolism (Figure 1).

**Figure 2:**
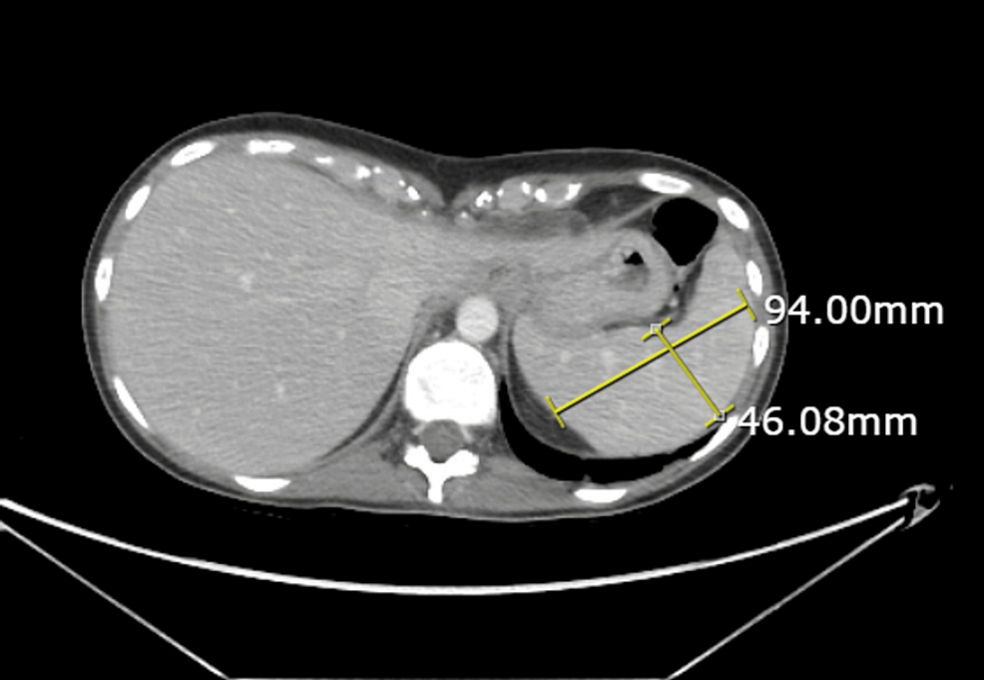
CT abdomen showing enlarged spleen

**Figure 3:**
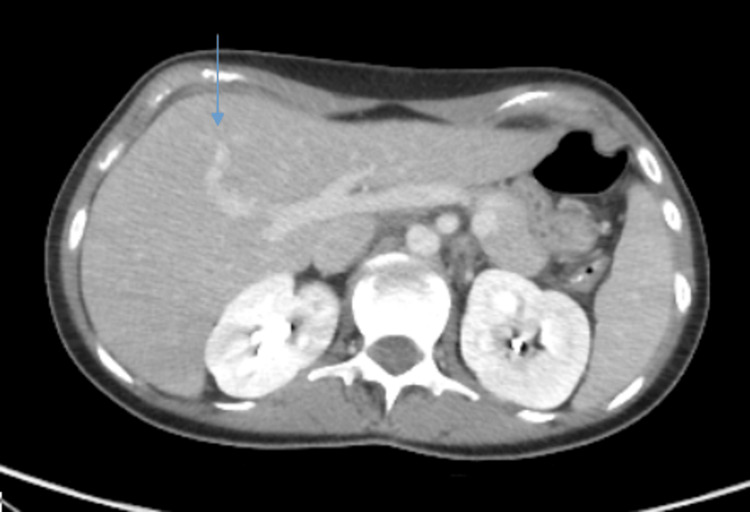
CT scan of the abdomen showing enlarged liver

CT abdomen and pelvis showed mild splenomegaly (volume of 370 mL) (Figure 2) and hepatomegaly, with the liver measuring 24.5 cm in the greatest craniocaudal dimension (Figure 3).

During the hospital course, blood cultures were negative two times, indicating no blood infection. Any active infection that could have caused her symptoms was excluded. HIV, hepatitis antibodies, cytomegalovirus, Epstein-Barr virus (EBV), and herpes antibodies were negative. The Mono spot test was negative as well. The fibrinogen level was decreased (less than 150mg/dL). Workup showed antinuclear antibody screen +, dsDNA negative, Jo-1 Ab negative, Sjogren’s Ab SS-A and SS-b negative, and Histone Ab negative (Tables 1, 2).

**Table 1:** Results of the autoimmune workup performed during admission ANA: Antinuclear antibody

Test	Result	Reference range
ANA screen	Positive	Positive
DNA (DS) ABS	1	negative < or equal to 4; indeterminate 5-9; positive > or = 10
JO-1 AB	<1	<1 negative
SJOGREN’S AB (SS-A)	<1	<1 negative
SJOGREN’S AB (SS-B)	<1	<1 negative
HISTONE AB	<1	<1 negative

**Table 2:** IL2-R alpha, used to diagnose HLH in symptomatic patients, was also elevated

	Result	Reference
IL2 RALPHA (CD25) soluble	3711 pg/ml	532-1891

Based on the above findings, and elevated IL-2, HLH was suspected to be patient fulfilled as the patient met five out of eight criteria of HLH-2004.

She received 10 mg of dexamethasone and ketorolac in the emergency department. She was started on antibiotics and was admitted for further management. During the hospital course, she was started on prednisone and continued cyclosporine 100 mg two times a day. The patient was treated with cyclosporine and prednisone for five days and improved. Lab trends throughout the hospital course were as follows (Table 3).

**Table 3:** Trend of lab values throughout the hospital admission AST: Serum aspartate aminotransferase; ALT: Alanine aminotransferase; CRP: C-reactive protein

	Reference range	Day 1	Day 2	Day 3	Day 4	Day 5	Day 6
Hemoglobin (gm/dl)	12-16 g/dl	11.5	10.7	10.7	10.2	11.8	11
White blood count (per microliters)	4.5 to 11.0 × 109/L	29.8	26.8	19.4	11.2	11.7	10
Platelets (10 per microliter)	150,000 to 450,000/microliter	431	427	344	420	406	483
AST (U/L)	8-33 U/L	22	19	22	23	14	15
ALT (U/L)	7 to 56 U/L	13	13	18	25	27	28
Creatinine (mg/dl)	0.59 to 1.04 mg/dL	0.7	0.8	0.6	0.6	0.7	0.6
Ferritin (ng/ml)	12 to 150 ng/mL	3083	3257	4206	3392	2260	1433
CRP	<0.3 mg/dL	312.05	294	257.27	128.73	122.98	67.95

She was discharged with follow-up appointments made for the heme/onc outpatient clinic.

## Discussion

HLH is a challenging disease to diagnose and treat as it can present in different ways. HLH is challenging to diagnose and treat as it can present in different ways. HLH can be triggered by events disrupting immune homeostasis, including common infections, and may occur as a familial or sporadic disorder. Both those with a genetic predisposition and those with sporadic cases can develop the condition as a result of infection. Genetic defects have a significant impact on childhood HLH and are also increasingly found in adult cases [4-6]. With genetic information, it becomes possible to determine the likelihood of recurrence of the disease and the necessity of a hematopoietic cell transplant.

Primary vs. secondary have been removed from as infections or other immune activating events can trigger both primary and secondary HLH and gene mutations can be found in individuals of any age and with any family history.

According to the HLH-2004, five out of eight criteria must be fulfilled to meet the diagnosis of HLH, including fever, bi-cytopenia, hypertriglyceridemia, splenomegaly and/or hypofibrinogenemia, hemophagocytosis, low/absent NK-cell-activity, hyper-ferritinemia, and high-soluble interleukin-2-receptor levels (Table 4) [7].

**Table 4:** Criteria for diagnosis of HLH Meet five of the eight criteria for diagnosis of HLH. HLH: Hemophagocytic lymphohistiocytosis; NK: Natural killer; IL2: Interleukin-2

Serial No	Criteria
1	Fever
2	Splenomegaly
3	Cytopenia (affecting >2 or equal lineages in peripheral blood)
4	Hypertriglyceridemia and/or hypofibrinogenemia: fasting triglycerides greater than or equal to 265 mg/dl, Fibrinogen less than or equal to <150 gm/L
5	Hemophagocytosis in bone marrow, spleen, or lymph nodes (no evidence of malignancy)
6	Ferritin greater than or equal to 500 ug/L
7	Soluble IL2 receptor greater than or equal to 2400 U/ml
8	Low or absent NK activity

HLH is an immune response that can lead to multiorgan failure. HLH secondary to vaccines is not common, but it has been reported to occur after the influenza vaccine [8,9].

COVID-19 vaccine has been reported to cause immune-related diseases such as cytokine storm syndrome. It can induce the production of certain cytokines, and those excessive cytokines can act as a trigger for HLH [10]. Our patient did not have a history of any autoimmune disease and presented with fever and systemic symptoms of HLH after the COVID vaccine; the trigger of HLH in our patient was likely the COVID-19 vaccine, as her symptoms started shortly after she received her first dose.

While the exact mechanisms underlying COVID-19 vaccination-associated HLH are not yet understood, it is hypothesized that the extensive immune activation induced by vaccines could instigate a pronounced cytokine storm, potentially contributing to the development of HLH. HLH arises from a positive feedback loop involving macrophages, activated CD8+ T cells, and antigen-presenting cells, which stimulate each other, causing a "cytokine storm." It is also thought that the secretion of interleukin one beta (IL-1-beta) from macrophages in response to the spike protein produced by mRNA vaccines is responsible for the development of hyperinflammatory syndrome post-SARS-CoV-2 mRNA vaccination [11].

Common laboratory findings found in patients with HLH include elevated inflammatory markers, elevated ferritin, hypertriglyceridemia, cytopenias, and abnormal liver function tests. Patients also have elevated CD25, which is a marker for increased activity of lymphocytes [12]. Elevated ferritin is a response to the increased inflammatory reaction going on in the body. Although commonly elevated in patients with HLH, as seen in our case, elevated ferritin is neither specific nor sensitive for the diagnosis of HLH [13].

Treatment of COVID-19 vaccine-induced HLH involves overcoming the immune and hyperinflammatory response. Initially, glucocorticoids are always included in the treatment of HLH. Immunosuppressants were added in patients who were resistant to steroids [14]. Rituximab and IV immunoglobulins are used in the treatment of viral-induced HLH, such as EBV-triggered HLH. The standard of care includes the use of etoposide, which helps kill the lymphocytes. Cyclosporine inhibits the synthesis of interleukins and decreases T-cell activation and differentiation. If the central nervous system is involved, intrathecal methotrexate and steroids can be used. As used in our patients, treatment with steroids and cyclosporine usually helps in dramatic improvement [15]. Remission can be achieved by the use of immunosuppressive drugs with steroids, followed by cyclosporine. Patients with COVID-19-associated HLH showed a positive response to treatment with Anakinra [16].

Response to treatment is measured by the resolution of the inflammatory markers and clinical improvement in symptoms. In this patient, the ferritin and other inflammatory markers started trending down, and she improved clinically.

Very few cases have been reported in literature where HLH has been triggered by the COVID-19 vaccine [10,11,17]. This report should not be viewed as a reason to avoid vaccination. However, it is to raise awareness among physicians about the possibility of such conditions after vaccines in patients who have underlying inflammatory conditions for prompt diagnosis and treatment.

Our case report has certain limitations as well. Background factors are influential in patients predisposed to developing cytokine storms following SARS-CoV-2 vaccination. Moreover, the mechanism by which cytokine storms precipitate HLH, in cases like the one described, particularly in individuals lacking a history of autoimmune diseases or allergies, remains unclear. While there appears to be a temporal association between COVID-19 vaccination and the onset of HLH in our patient, establishing a direct causal relationship requires further investigation.

## Conclusions

In conclusion, HLH is a severe disorder that can quickly lead to organ failure. Suspicion of HLH should lead to an immediate investigation of the underlying cause, including the history of vaccination. Although rare, it can lead to it, as seen in our case. Early recognition and treatment will lead to increased chances of improvement and survival.
